# The use and potential impact of digital health tools at the community level: results from a multi-country survey of community health workers

**DOI:** 10.1186/s12889-024-18062-3

**Published:** 2024-03-01

**Authors:** Courtney T. Blondino, Alex Knoepflmacher, Ingrid Johnson, Cameron Fox, Lorna Friedman

**Affiliations:** 1 Department of Health Studies, School of Arts and Sciences, University of Richmond, Richmond, VA 23173 USA; 2 Mercer, New York, NY 10036 USA; 3 Platform for Shaping the Future of Health & Healthcare, World Economic Forum, New York, NY 10017 USA

**Keywords:** Digital health, Community health workers, Technology, Global

## Abstract

**Background:**

Community health workers (CHWs) are increasingly viewed as a critical workforce to address health system strengthening and sustainable development goals. Optimizing and widening the capacity of this workforce through digital technology is currently underway, though there is skepticism regarding CHWs’ willingness and optimism to engage in digital health. We sought to understand CHWs’ perceptions on the use of digital health tools in their work.

**Methods:**

We obtained survey data from 1,141 CHWs from 28 countries with complete study information. We conducted regression analyses to explore the relationship between CHWs’ training and perceived barriers to digital health access with current use of digital devices/tools and belief in digital impact while adjusting for demographic factors.

**Results:**

Most of the CHWs worked in Kenya (*n* = 502, 44%) followed by the Philippines (*n* = 308, 27%), Ghana (*n* = 107, 9.4%), and the United States (*n* = 70, 6.1%). There were significant, positive associations between digital tools training and digital device/tool use (Adjusted Odds Ratio (AOR) = 2.92, 95% CI = 2.09–4.13) and belief in digital impact (AOR_high impact =_ 3.03, 95% CI = 2.04–4.49). CHWs were significantly less likely to use digital devices for their work if they identified cost as a perceived barrier (AOR_mobile service cost =_ 0.68, 95% CI = 0.49–0.95; AOR_phone/device cost =_ 0.66, 95% CI = 0.47–0.92). CHWs who were optimistic about digital health, were early adopters of technology in their personal lives, and found great value in their work believed digital health helped them to have greater impact. Older age and greater tenure were associated with digital device/tool use and belief in digital impact, respectively.

**Conclusions:**

CHWs are not an obstacle to digital health adoption or use. CHWs believe that digital tools can help them have more impact in their communities regardless of perceived barriers. However, cost is a barrier to digital device/tool use; potential solutions to cost constraints of technological access will benefit from further exploration of reimbursement models. Digital health tools have the potential to increase CHW capacity and shape the future of community health work.

**Supplementary Information:**

The online version contains supplementary material available at 10.1186/s12889-024-18062-3.

## Background

Community health workers or CHWs (also known as agents, navigators, health coaches, health educators, health outreach workers, public health aids, caregivers, etc.) are trusted and familiar members of a community who are trained to deal with the varied health problems of people within their community [1–4]. The World Health Organization and others have advocated for optimizing and expanding the capacity of CHWs as a path to Universal Health Coverage and as a strategy to address health related Sustainable Development Goals [5]. This is especially so in low- and middle-income countries (LMICs) where there is a projected shortfall of 10 million health workers by 2030 [2, 6, 7]. Digital health, or the use of a wide range of digital technologies and devices for health [8], is being explored to expand the activities and impact of CHWs in multiple settings. Currently, CHWs are equipped with mobile phones, tablets, and other devices that support their daily activities. They engage with a growing digital ecosystem to support them in screening, managing, and treating a variety of health conditions [9, 10]. Many CHWs use digital health technologies to effectively tackle health challenges including but not limited to non-communicable diseases (NCDs), maternal and child health, and pandemic preparedness and response [11–13].

Several effective digital models, specifically those targeting NCDs, couple technology with CHWs using a multi-stakeholder approach with private–public collaboration. For example, the *Digital Lifecare* model provides a modern digital platform to health workers across the Indian health system with mobile, cloud, and analytics applications so that they may screen, diagnose, manage, and track NCDs, providing a unified view of patients over time through secure data sharing [14]. Initiatives can be implemented even with limited internet access. For instance, the *Padayon digital health* model for diabetes and hypertension equips CHWs with an offline-first platform designed to address the challenge of limited connectivity across LMICs [11]. CHWs leverage the offline-first mobile health app to provide subscribed members in the Philippines with blood pressure and random blood sugar testing and prescribed medicine monitoring, ultimately improving blood pressure by 29% and blood sugar level by 8% in the community compared to baseline [11].

Despite these and other models demonstrating efficacy within their respective communities, concerns about barriers to CHWs’ digital health use remain. Recent studies identified challenges related to funding, health literacy of CHWs, resistance to change of existing behaviors and attitudes, and CHWs’ lack of adequate supplies [9, 15, 16]. Regardless, peer-reviewed published literature consistently demonstrates an improvement in health outcomes when CHWs are involved in the delivery of care given their competence as well as the social capital they have with their community [17–23]. Therefore, barriers to the acceptance, engagement, and impact of digital health technologies at the community level may be mitigated by the involvement of CHWs.

We sought to understand the perspective and current engagement of CHWs’ use of digital technologies to support and facilitate their regular healthcare activities. Specifically, we tested the relationship between CHWs’ training and perceived barriers to digital health access in their communities with their current use of digital devices and belief in digital impact. The results of this study will inform current and future CHW models to combat a variety of health conditions across multiple countries.

## Methods

### Study population

Community health workers from across the globe were surveyed between November 2022 and May 2023. The Digital-Temple Model which demonstrates the impact of digitalization on health systems in relation to NCDs involving complex interactions among patients, providers, and CHWs informed the questionnaire development [9]. The model posits that digitization is the catalyst in the system and the impact of patient-provider-CHW interactions influences the expansion of access and quality services, training and supervision of personnel, and research and evaluation [9].

The online survey was written in English using the Qualtrics Experience Management (XM) platform and translated by native speakers into eleven languages including Arabic, Bengali, French, Hiligaynon, Hindi, Mandarin, Nepali, Portuguese, Spanish, Swahili, and Tamil to reduce the language barrier of participation. Minor modifications were made to the questionnaire as translators advised on differences in the language translation that would result in confusion or were not culturally appropriate. The survey was distributed via email to leaders of approximately 40 international organizations and 70 academic institutions that work with and train CHWs. These organizations and institutions were selected from desktop and literature review. The email requested the online survey be disseminated to CHWs and encouraged a snowball sampling approach where the CHWs could share the survey with other CHWs within their professional network. Up to four reminder emails were sent over the course of the data collection period to encourage survey participation. Confirmation of email receipt and execution of survey distribution occurred from all international organizations and only ten of the academic institutions. Survey respondents were notified and consented to their data being collected anonymously (no identifiable information was collected) with results being released only in aggregate.

A total of 1,664 participants responded to the survey within the six-month data collection period. Those with missing data were excluded from the analytic sample, resulting in a final sample of 1,141 respondents representing 28 countries. Of the 523 respondents that did not complete the survey, at least 67% of data were missing on variables tested in this study. This missingness is likely due to participants opening the survey but not finishing all questions for reasons that are unclear. Specifically, 30 respondents made 0% survey progress (opened survey but did not select any response options), 329 respondents made 19% progress, 79 respondents made 43% progress, 47 respondents made 57% progress, and 38 respondents made 67% progress. These progress points (i.e., 19%, 43%, 57%, and 67%) correspond to section breaks in the survey.

### Measures

#### Current digital use and belief in digital impact

Two major outcomes of interest were studied: current digital use and belief in how digital applications would help CHWs have more impact in their communities. CHWs responded to a binary (yes/no) question about their current use of digital technologies (“Do you currently use digital devices like smartphones, tablets, etc. when providing services to your community as a health worker?). CHWs also responded to a categorical item “How would a digital application help you have more impact?” with eight response options including: 1) improved and faster data collection, 2) easy access to health education, 3) diagnosis of illness, 4) management of chronic conditions at home, 5) improve population/community health, 6) reduce errors and/or duplication in paper based records, 7) more frequent contact with community members without travel, and 8) more frequent contact with community health staff, clinical pharmacists without travel. These items were informed by the 12 core functions of the Digital-Temple Model [9] with expert consultation from CHWs and CHW thought leaders to contextualize the final eight items. CHWs were able to select as many response options as applied. To use this variable in a regression model, it was first transformed into a sum score (ordinal variable ranging from 0–8) then further re-categorized into low (0–2), moderate (3–5), and high (6–8) impact levels as the variable did not follow a normal distribution and for ease of interpretability. These cut points were identified based on the distribution of the data in the sample: N_low_ = 303 (26.6%), N_moderate_ = 474 (41.5%), N_high_ = 364 (31.9%).

#### Training

CHWs were asked, “What kind of training have you received as a community health worker?” with 18 response options. A sum score was created by totaling the number of trainings, resulting in an ordinal variable ranging from 0–18. A binary variable (yes/no) for digital tools training (e.g., computer, tablets, smart phones, etc.) was further analyzed in the regression models.

#### Barriers

As advocates for their communities, CHWs answered a question related to barriers: “What are the barriers that individuals in your community face in accessing digital health?” with eight response options including: 1) limited or no internet connectivity, 2) limited or no electricity/power, 3) cost of mobile phone services, 4) cost of internet services, 5) cost of phone/device, 6) limited experience with technology, 7) prefer traditional face-to-face interaction, and 8) distrust technology. A sum score was created by totaling the number of barriers a CHW selected, resulting in an ordinal variable ranging from 0–8.

#### Other covariates

Other covariates were included in the regression modeling to understand their relationship with the outcome variables. CHWs were asked “What do you value most about your work?” and were permitted to select up to three response options out of the following five in order to determine value priorities: 1) working with community members/community engagement, 2) teaching others and helping individuals get healthy, 3) expanding my skills, 4) getting peer support from the other community health workers, and 5) supplementing my income. A sum score was created by totaling the number of values a CHW selected, resulting in an ordinal variable ranging from 0–3 as CHWs were only permitted to select up to three values. CHWs shared their optimism of digital health in their communities (“How optimistic are you that digital health can have a positive impact in the community you serve?”) with five response options: Very optimistic, Optimistic, Neutral, Not optimistic, or Not at all optimistic. CHWs also shared their level of digital adoption in their personal life by classifying themselves within one of three categories: 1) I am often one of the first people to try new technology and devices, 2) I often wait for others to try new technology and devices first, and 3) I am often the last person to try new technology and devices. CHWs were asked the geographic representation of the population with which they work (3 levels: Urban, Suburban, Rural) as well as their tenure as a CHW (3 levels: Less than 1 year, 1 to 5 years, More than 5 years). Three demographic questions were asked of all survey respondents: age, gender identity, and country in which you work. Age was a six-level categorical variable (18–24 years, 25–34 years, 35–44 years, 45–54 years, 55–64 years, 65 years and over). Gender identity was a four-level categorical variable (male, female, non-binary, prefer not to say). These categories are not inclusive of all potential gender identities. Minimal options were provided to effectively translate across the eleven languages. Country in which you work was provided as a drop-down selection. A race/ethnicity/origin question was asked only of CHWs who indicated they worked in the United States. This was a ten-level categorical variable (White, Black or African American, Hispanic or Latino or of Spanish origin, Asian, Middle Eastern, Native (Indigenous) American/Alaskan, Native Hawaiian or Native Pacific Islander, Other, Don’t Know, Prefer not to answer).

### Statistical analysis

Descriptive statistics (categorical: N, %; continuous: mean, range, and standard deviation [SD]) were provided across all variables for the total sample. Chi-square and T-tests were used to test for significant differences between outcomes for each variable. Given that 44% and 27% of the sample worked in Kenya and the Philippines, respectively, we tested for significant differences across these countries only, as the purpose of this study was not to understand significant differences in CHW perceptions across countries. Stratified country regression results for Kenya and the Philippines are provided in Supplementary Material. For the total sample accounting for all countries, unadjusted logistic regression was used to test the association between training and barriers with current digital use (binary: yes/no) when providing services to the community as a health worker. Unadjusted multinomial regression was used to test the association between training and barriers with belief in digital impact (categorial: low, moderate, high). Ordinal regression was not used for the digital impact model as the proportional odds assumption was violated. Statistically significant associations identified in the unadjusted models were used for the adjusted models. Odds ratios (OR) or adjusted odds ratios (AOR) and 95% confidence intervals (95% CI), profiled from estimates of standard error, are reported. Data management and all statistical analyses were performed in R, Version 4.2.2 [24].

## Results

### Descriptive statistics for the total sample

Data from 1,141 participants with complete information were analyzed. The majority of CHWs were women (78.4%) of middle age (25–34 years: 27.6%; 35–44 years: 31.1%; 45–54 years: 24.6%). Twenty-eight countries were represented with the most participants working in Kenya (*n* = 502, 44%) followed by the Philippines (*n* = 308, 27%), Ghana (*n* = 107, 9.4%), and the United States (*n* = 70, 6.1%) (Table 1).

**Table 1:** Descriptive statistics of community health worker demographics

	**Total**	**Kenya**	**Philippines**
	1141 (100%)	502 (44%)	308 (27%)
**Gender identity**^**a**^			
Male	225 (19.7)	122 (24.3)	6 (1.9)
Female	895 (78.4)	366 (72.9)	298 (96.8)
Non-binary/prefer not to say	21 (1.8)	14 (2.8)	4 (1.3)
**Age**^**a**^			
18–24 years	36 (3.2)	14 (2.8)	6 (1.9)
25–34 years	315 (27.6)	86 (17.1)	64 (20.8)
35–44 years	355 (31.1)	164 (32.7)	110 (35.7)
45–54 years	281 (24.6)	163 (32.5)	82 (26.6)
55–64 years	117 (10.3)	60 (12.0)	36 (11.7)
65 years and older	37 (3.2)	15 (3.0)	10 (3.2)
**Country**			
Albania	2 (0.02)		
Algeria	1 (0.01)		
Angola	1 (0.01)		
Argentina	1 (0.01)		
Australia	1 (0.01)		
Austria	1 (0.01)		
Brazil	1 (0.01)		
China	1 (0.01)		
Columbia	10 (0.09)		
Ghana	107 (9.4)		
Guinea	2 (0.02)		
Iceland	1 (0.01)		
India	37 (3.2)		
Italy	1 (0.01)		
Jordan	1 (0.01)		
Kenya	502 (44.0)		
Lesotho	6 (0.05)		
Liberia	1 (0.01)		
Malawi	6 (0.05)		
Mexico	6 (0.05)		
Nepal	3 (0.03)		
Nigeria	2 (0.02)		
Philippines	308 (27.0)		
Samoa	1 (0.01)		
Sierra Leone	20 (1.8)		
Tanzania	37 (3.2)		
Uganda	11 (1.0)		
United States of America	70 (6.1)		

Racial identity was only asked of CHW who specified that they worked in the United States and were categorized as follows: Asian (1, 1.4%), Black or African American (21, 30%), Hispanic or Latino or of Spanish origin (10, 14.3%), Native (Indigenous) American/Alaskan (1, 1.4%), White (32, 45.7%), Prefer not to answer (3, 4.3%), Other (2, 2.9%)

^a^Indicates significant differences (*p*-value < 0.05) across Kenya and the Philippines

CHWs primarily worked in rural settings (61.8%) with a tenure of more than five years (60.5%). On average, CHWs engaged 7.3 trainings (range = 0–18; SD = 5.0). Approximately, 77% of the sample had received training in NCD care. Other most commonly reported trainings included health education methods (69.3%), sanitation and hygiene promotion and education (65.3%), and data collection (56.5%). CHWs provided a mean of 6.96 services (range = 0–17, SD = 4.2) and most commonly reported blood pressure check/monitoring (66.1%), sanitation and hygiene promotion and education (64.7%), and data collection and community health needs assessment (64.2%). CHWs primarily valued working with community members (88.4%), teaching others and helping individuals get healthy (82.6%), and expanding their skills (60.6%) (Table 2).

**Table 2:** Descriptive statistics of community health work

	**Total**	**Kenya**	**Philippines**
	N (%)	N (%)	N (%)
**Geographic representation of population**^**a**^			
Urban	352 (30.9)	157 (31.3)	97 (31.5)
Suburban	84 (7.4)	37 (7.4)	6 (1.9)
Rural	705 (61.8)	308 (61.4)	205 (66.6)
**CHW tenure**^**a**^			
Less than 1 year	85 (7.4)	14 (2.8)	19 (6.2)
1–5 years	366 (32.1)	104 (20.7)	117 (38.0)
More than 5 years	690 (60.5)	384 (76.5)	172 (55.8)
**Training received as a CHW**			
*Sum of trainings (mean, sd)*^*a*^	*7.31 (5.01)*	*8.39 (4.91)*	*5.15 (4.13)*
NCD (chronic health conditions like hypertension (high blood pressure), diabetes, cardiovascular disease, and/or stroke)	878 (77.0)	322 (64.1)	287 (93.2)
First Aid/CPR	485 (42.5)	182 (36.3)	166 (53.9)
Digital tools (e.g., computer, tablets, smart phones, etc.)	492 (43.1)	213 (42.4)	91 (29.5)
Sanitation and hygiene promotion and education	745 (65.3)	389 (77.5)	184 (59.7)
Health education methods	791 (69.3)	366 (72.9)	210 (68.2)
Individual health assessment skills	487 (42.7)	248 (49.4)	88 (28.6)
Interviewing techniques	295 (25.9)	105 (20.9)	61 (19.8)
Counseling/coaching techniques	456 (40.0)	232 (46.2)	64 (20.8)
Care coordination	273 (23.9)	123 (24.5)	46 (14.9)
Making referrals	646 (56.6)	392 (78.1)	111 (36.0)
Case management	429 (37.6)	276 (55.0)	21 (6.8)
Health equity	310 (27.2)	146 (29.1)	45 (14.6)
Cultural competency	181 (15.9)	77 (15.3)	16 (5.2)
Building trust and relationships within a community	546 (47.9)	292 (58.2)	81 (26.3)
Community organizing/advocacy	501 (43.9)	288 (57.4)	77 (25.0)
Community health assessment skills	570 (50.0)	295 (58.8)	119 (38.6)
Health system navigation	213 (18.7)	107 (21.3)	35 (11.4)
Data collection	649 (56.9)	346 (68.9)	130 (42.2)
Evaluation and research skills	275 (24.1)	137 (27.3)	42 (13.6)
**Value most about your work**			
*Sum of values (mean, sd)*^*a*^	*2.66 (0.66)*	*2.76 (0.55)*	*2.53 (0.75)*
Working with community members/community engagement	1009 (88.4)	476 (94.8)	252 (81.8)
Teaching others and helping individuals get healthy	943 (82.6)	424 (84.5)	244 (79.2)
Expanding my skills	692 (60.6)	331 (65.9)	165 (53.6)
Getting peer support from other community health workers	309 (27.1)	131 (26.1)	94 (30.5)
Supplementing my income	79 (6.9)	26 (5.2)	24 (7.8)

CHWs were allowed to select up to three response options, out of five, for the “value most about work” item

^a^Indicates significant differences (*p*-value < 0.05) across Kenya and the Philippines

80.2% of the sample currently use digital devices and tools for their work and, of those, the majority utilized a mobile device/smartphone (74.8%). There was a mean of 2.9 barriers to digital access identified by the CHWs for individuals in their community (range = 0–8, SD = 1.9). The most commonly reported barriers were limited or no internet connectivity (60.4%), cost of internet services (52.6%), cost of mobile phone services (47.9%), and cost of phone/device (40.1%). At lower levels, CHWs also identified limited experience with technology/not knowing how to use technology (34.7%), prefer traditional face-to-face interaction (25.8%), and distrust in technology (8.3%) as barriers to digital access for people in their community. CHWs believed that digital would help them have more impact (mean = 4.25, range = 0–8, SD = 2.30) specifically with improved and faster data collection (85.1%), easy access to health education (73.9%), and more frequent contact with community members without travel (53.0%). Belief in digital impact was well distributed across the low (26.6%), moderate (41.5%), and high (31.9%) levels. Overall, the sample was either very optimistic (55.3%) or optimistic (28.8%) that digital health can have a positive impact in the community. CHWs also identified themselves as often one of the first people to try new technology and devices (74.5%) compared to waiting for others to try (20.8%) or being the last person to try (4.6%) new technology and devices first (Table 3).

**Table 3:** Descriptive statistics of use, engagement, and perceptions of digital technologies

	**Total**	**Kenya**	**Philippines**
	N (%)	N (%)	N (%)
**Current use of digital devices when providing services to your community as a health worker**^**a**^			
Yes	915 (80.2)	352 (70.1)	268 (87.0)
No	226 (19.8)	150 (29.9)	40 (13.0)
**Device/tool type**^a^			
*Total number of devices/tools used (mean, sd)*	*1.32 (1.14)*	*1.06 (1.14)*	*1.12 (0.78)*
Mobile device/smartphone	853 (74.8)	341 (67.9)	262 (85.1)
Tablet	210 (18.4)	44 (8.8)	11 (3.6)
Electronic medical record	129 (11.3)	21 (4.2)	15 (4.9)
Health risk assessment on an internet application/mobile health platform	168 (14.7)	70 (13.9)	36 (11.7)
Remote sensing and wearables	64 (5.6)	12 (2.4)	12 (3.9)
Decision support systems/apps that help me make decisions	87 (7.6)	43 (8.6)	8 (2.6)
**Barriers to digital access for individuals in your community**			
*Sum of barriers (mean, sd)*^*a*^	*2.94 (1.90)*	*3.34 (1.84)*	*2.21 (1.50)*
Limited or no internet connectivity	689 (60.4)	270 (53.8)	210 (68.2)
Limited or no electricity/power source	274 (24.0)	177 (35.3)	19 (6.2)
Cost of mobile phone services	546 (47.9)	298 (59.4)	122 (39.6)
Cost of internet services	600 (52.6)	314 (62.5)	105 (34.1)
Cost of phone/device	457 (40.1)	258 (51.4)	86 (27.9)
Limited experience with technology/not knowing how to use technology	396 (34.7)	192 (38.2)	74 (24.0)
Prefer traditional face-to-face interaction	294 (25.8)	124 (24.7)	63 (20.5)
Distrust in technology	95 (8.3)	44 (8.8)	1 (0.3)
No particular barriers	34 (3.0)	7 (1.4)	5 (1.6)
**Belief in digital impact**			
*Sum of impact (mean, sd)*^*a*^	*4.25 (2.30)*	*4.61 (2.13)*	*3.69 (2.38)*
Belief in digital impact			
Low (0–2)	303 (26.6)	95 (18.9)	120 (39.0)
Moderate (3–5)	474 (41.5)	232 (46.2)	108 (35.1)
High (6–8)	364 (31.9)	175 (34.9)	80 (26.0)
Improved and faster data collection	971 (85.1)	456 (90.8)	249 (80.8)
Easy access to health education	843 (73.9)	396 (78.9)	214 (69.5)
Diagnosis of illness	347 (30.4)	130 (25.9)	78 (25.3)
Management of chronic conditions at home	438 (38.4)	219 (43.6)	82 (26.6)
Improve population/ community health	515 (45.1)	219 (43.6)	142 (46.1)
Reduce errors and/or duplication in paper based records	594 (52.1)	326 (64.9)	101 (32.8)
More frequent contact with community members without travel	605 (53.0)	288 (57.4)	154 (50.0)
More frequent contact with community health staff, clinical, pharmacists without travel	533 (46.6)	278 (55.4)	116 (37.7)
I already use many forms of digital health in my work, and it is helpful	329 (28.8)	133 (26.5)	84 (27.3)
I already use many forms of digital health in my work, and it is not especially helpful	78 (6.8)	28 (5.6)	25 (8.1)
**Optimism that digital health can have a positive impact in the community**^**a**^			
Very optimistic	631 (55.3)	335 (66.7)	108 (35.1)
Optimistic	329 (28.8)	101 (20.1)	130 (42.2)
Neutral	148 (13.0)	52 (10.4)	62 (20.1)
Not optimistic	15 (1.3)	6 (1.2)	2 (0.6)
Not at all optimistic	18 (1.6)	8 (1.6)	6 (1.9)
**In my personal life:**^**a**^			
I am often one of the first people to try new technology and devices	852 (74.7)	425 (84.7)	199 (64.6)
I often wait for others to try new technology and devices first	237 (20.8)	51 (10.2)	92 (29.9)
I am often the last person to try new technology and devices	52 (4.6)	26 (5.2)	17 (5.5)

^a^Indicates significant differences (*p*-value < 0.05) across Kenya and the Philippines

### Descriptive statistics for Kenya and the Philippines

The Philippines had significantly fewer men than women (Philippines = 1.9%, Kenya = 24.3%, *p* < 0.05). The total sample had significantly younger respondents compared to Kenya and the Philippines, specifically under the age of 35 years (Table 1). CHWs in the Philippines worked significantly less in suburban settings compared to CHWs in Kenya and the total sample (Philippines = 1.9%, Kenya = 7.4%, total = 7.4%, *p* < 0.05). CHWs in Kenya were significantly more tenured (greater than 5 years = 76.5%), had a greater number of trainings (mean = 8.4) but had significantly less engaging in NCD training (64.1%) and selected more values about their work as CHWs (mean = 2.8) compared to CHWs in the Philippines or the total sample. In the Philippines, CHWs had significantly less training in digital tools (Table 2). CHWs in the Philippines significantly used more digital devices, specifically mobile devices/smartphones compared to Kenya (Philippines = 85.1%, Kenya = 67.9%, *p* < 0.05). CHWs in Kenya identified significantly more barriers but also endorsed greater belief in digital impact compared to the Philippines and the total sample. Kenya also had significantly more people who were very optimistic that digital can have a positive impact in the community (Kenya = 66.7%, total = 55.3%, Philippines = 35.1%, *p* < 0.05) and more CHWs who were often the first people to try new technology and devices (Kenya = 84.7%, total = 74.7%, Philippines = 64.6%, *p* < 0.05) (Table 3).

### Outcome #1: current digital use

#### Unadjusted logistic regression

There was no association between the number of trainings received as a CHW and current digital use for the total analytic sample (OR = 1.02, 95% CI = 0.99–1.05). However, there was a significant association between digital tools training and current digital use (OR = 2.84, 95% CI = 2.06–3.98). There was no association between the number of barriers and current digital use yet there were three specific types of barriers that had a statistically significant association with current digital use: having limited or no internet connectivity (OR = 1.62, 95% CI = 1.21–2.18), cost of a mobile phone device (OR = 0.67, 95% CI = 0.50–0.90), and cost of mobile phone service (OR = 0.62, 95% CI = 0.46–0.83). None of the demographic variables were significantly associated with current digital use except for the oldest age category (65 years and older) though the confidence range was not precise (Table 4).

**Table 4:** Summary of unadjusted and adjusted associations with current digital use of the total sample (*N* = 1141)

	OR (95% CI)	AOR (95% CI)
*CHW trainings (sum)*	1.02 (0.99–1.05)	–
Digital tools training	**2.84 (2.06–3.98)**	**2.92 (2.09–4.13)**
*Barriers to the community (sum)*	0.97 (0.90–1.04)	–
Limited or no internet connectivity	**1.62 (1.21–2.18)**	**1.62 (1.19–2.20)**
Limited or no electricity/power source	1.04 (0.74–1.47)	–
Cost of mobile phone services	**0.62 (0.46–0.83)**	**0.68 (0.49–0.95)**
Cost of internet services	1.04 (0.78–1.39)	–
Cost of phone/device	**0.67 (0.50–0.90)**	**0.66 (0.47–0.92)**
Limited experience with technology/not knowing how to use technology	0.81 (0.60–1.10)	–
Prefer traditional face-to-face interaction	1.07 (0.77–1.50)	–
Distrust in technology	0.92 (0.56–1.58)	–
No particular barriers	1.16 (0.51–3.13)	–
Age (ref = 18–24 years)		
25–34 years	2.04 (0.89–4.38)	1.86 (0.79–4.14)
35–44 years	1.39 (0.61–2.92)	1.41 (0.60–3.08)
45–54 years	1.33 (0.58–2.83)	1.39 (0.59–3.07)
55–64 years	1.57 (0.65–3.66)	1.63 (0.65–3.93)
65 years and over	**4.36 (1.19–20.92)**	**4.73 (1.26–23.21)**
Gender (ref = Prefer not to say)		–
Female	2.03 (0.63–5.78)	
Male	2.18 (0.65–6.48)	
Non-binary	1.00 (0.14–9.04)	
Geographic region (ref = Suburban)		–
Rural	0.93 (0.51–1.62)	
Urban	0.98 (0.52–1.76)	
CHW tenure (ref = Less than 1 year)		–
1–5 years	1.16 (0.60–2.13)	
More than 5 years	0.75 (0.40–1.30)	

Bolded values indicate statistical significance at the *p* < 0.05 level

“– “ represents a not significant relationship though the cell sizes were too small to accurately calculate an odds ratio

#### Adjusted logistic regression

Variables that were significantly associated with the outcome, current digital use, were included in the final adjusted model (Table 4). The association between digital tools training (AOR = 2.92, 95% CI = 2.09–4.13), limited or no internet connectivity (AOR = 1.62, 95% CI = 1.19–2.20), cost of mobile phone services (AOR = 0.68, 95% CI = 0.49–0.95) and cost of phone/device (AOR = 0.66, 95% CI = 0.47–0.92) remained statistically significant and in the same direction as identified in the unadjusted bivariate analysis. Furthermore, age was only significantly associated with current digital use for the oldest age category (AOR = 4.73, 95% CI = 1.26–23.21) relative to the youngest age category (18–24 years).

### Outcome #2: belief in digital impact

#### Unadjusted multinomial regression

Compared to low belief in digital impact, the number of CHW trainings were significantly associated with moderate (OR = 1.19, 95% CI = 1.14–1.23) and high (OR = 1.34, 95% CI = 1.28–1.40) belief in digital impact. Specifically, digital tools training significantly increased the odds for moderate and high belief in digital impact (OR_moderate_ = 1.67, 95% CI = 1.22–2.28; OR_high =_ 3.92, 95% CI = 2.83–5.43) compared to low. Nevertheless, current use of digital tools was not significantly associated with belief in digital impact. The number of barriers in digital access were significantly associated with moderate and high belief in digital impact, compared to low, across the total sample (OR_moderate_ = 1.75, 95% CI = 1.56–1.96; OR_high =_ 2.35, 95% CI = 2.08–2.66). There was a positive, significant association between values about work and belief in digital impact at the moderate and high levels, compared to low (OR_moderate_ = 2.42, 95% CI = 1.96–3.00; OR_high =_ 4.18, 95% CI = 3.11–5.63). Relative to low impact, being optimistic (OR_moderate_ = 1.59, 95% CI = 1.03–2.47; OR_high =_ 2.27, 95% CI = 1.32–3.92) or very optimistic (OR_moderate_ = 2.32, 95% CI = 1.53–3.51; OR_high =_ 5.31, 95% CI = 3.19–8.85) to digital was significantly associated with moderate and high belief in digital impact compared to being neutral to digital. CHWs who identified as often being one of the first people to try new technology and devices, relative to CHWs who wait, had greater odds of believing in digital having moderate (OR = 1.72, 95% CI = 1.22–2.41) and high (OR = 2.31, 95% CI = 1.58–3.37) impact across the total sample (Table 5).

**Table 5:** Summary of unadjusted and adjusted associations with belief in digital impact of the total sample (*N* = 1141)

	**Moderate Impact** OR (95% CI)	**High Impact** OR (95% CI)	**Moderate Impact** AOR (95% CI)	**High Impact** AOR (95% CI)
*CHW trainings (sum)*	**1.19 (1.14–1.23)**	**1.34 (1.28–1.40)**	–	–
Digital tools training	**1.67 (1.22–2.28)**	**3.92 (2.83–5.43)**	**1.46 (1.03–2.07)**	**3.03 (2.04–4.49)**
Current digital use	0.96 (0.66–1.37)	1.00 (0.68–1.47)	–	–
*Barriers to the community (sum)*	**1.75 (1.56–1.96)**	**2.35 (2.08–2.66)**	**1.60 (1.42–1.80)**	**2.10 (1.85–2.39)**
*Value about work (sum)*	**2.42 (1.96–3.00)**	**4.18 (3.11–5.63)**	**2.03 (1.61–2.55)**	**3.07 (2.18–4.32)**
Optimism in digital (ref = neutral)				
Not at all optimistic	0.45 (0.15–1.36)	–	0.38 (0.12–1.22)	0.19 (0.02–1.75)
Not optimistic	0.95 (0.32–2.78)	–	0.92 (0.26–3.22)	–
Optimistic	**1.59 (1.03- 2.47)**	**2.27 (1.32–3.92)**	1.47 (0.90–2.40)	**2.08 (1.08–3.99)**
Very optimistic	**2.32 (1.53- 3.51)**	**5.31 (3.19–8.85)**	**1.88 (1.18–3.01)**	**3.87 (2.09–7.18)**
Digital adoption (ref = wait for others)				
First to try new tech	**1.72 (1.22–2.41)**	**2.31 (1.58–3.37)**	**1.75 (1.19–2.58)**	**2.27 (1.41–3.64)**
Last to try new tech	1.18 (0.58–2.43)	1.57 (0.73–3.39)	1.30 (0.58–2.91)	2.13 (0.82–5.58)
Age (ref = 18–24 years)			–	–
25–34 years	1.73 (0.49–1.83)	23.4 (3.06–179.04)		
35–44 years	1.93 (0.84–3.56)	21.0 (2.75–160.74)		
45–54 years	1.46 (0.95–3.93)	22.9 (2.99–175.45)		
55–64 years	2.03 (0.89–4.63)	35.3 (4.44–280.18)		
65 years and over	0.85 (0.31–2.32)	12.0 (1.38–104.79)		
Gender (ref = Prefer not to say)			–	–
Female	0.75 (0.18–3.01)	0.54 (0.13–2.20)		
Male	0.95 (0.23–3.96)	0.85 (0.20–3.54)		
Non-binary	1.00 (0.06–15.99)	1.50 (0.11–21.31)		
Geographic region (ref = Suburban)			–	–
Rural	1.02 (0.55–1.88)	1.17 (0.32–1.03)		
Urban	1.17 (0.62–2.21)	0.63 (0.34–1.18)		
CHW tenure (ref = Less than 1 year)				
1–5 years	**2.34 (1.38–3.98)**	**5.19 (2.54–10.59)**	**2.30 (1.26–4.19)**	**5.06 (2.15–10.19)**
More than 5 years	**2.61 (1.58–4.32)**	**5.60 (2.81–11.17)**	**2.22 (1.26–3.91)**	**4.25 (1.86–9.71)**

Reference for the outcome level was low impact (0–2). Bolded values indicate statistical significance at the *p* < 0.05 level

“– “ represents a not significant relationship though the cell sizes were too small to accurately calculate an odds ratio

#### Adjusted multinomial regression

Variables that were significantly associated with the outcome, belief in digital impact, were included in the final adjusted model (Table 5). All statistically significant associations identified in the unadjusted bivariate analysis for the total sample remained statistically significant though were attenuated. However, the association between being optimistic about digital at the moderate level for belief in digital impact relative to low impact (AOR_moderate_ = 1.47, 95% CI = 0.90–2.40) and being the first to try new technology at the moderate level relative to low impact (AOR_moderate =_ 1.75, 95% CI = 1.19–2.58) were the two exceptions.

## Discussion

This is one of the first studies that tested the association between training and perceived barriers to digital health access with current use of digital devices and belief in digital impact in a multi-country sample of CHWs. Our results demonstrate that CHWs are already using digital technologies, find digital to be valuable, and are optimistic about digital health technologies for the future. There were four major findings that warrant further discussion. First, digital tools training was a significant indicator for current use of digital devices as well as having greater belief in digital impact. Second, cost of both phones and mobile phone services was identified as a significant barrier to digital use in the community; however, regardless of the number of barriers identified, CHWs continued to greatly believe in digital impact. Third, CHWs who were optimistic, early adopters of digital in their personal lives, and found great value in their work had a greater belief in digital impact compared to counterparts without those attributes. Finally, older age and greater tenure were not barriers to digital usage; they were, in fact, indicators of digital device usage and belief in digital impact, respectively.

### Training and exposure to digital can increase its use and beliefs in digital impact

There was no significant association between the number of trainings and current use of digital devices, suggesting that more training does not necessarily indicate that a CHW would be more or less likely to use a digital device for their work. There was, however, a significant positive relationship with the number of trainings and belief in digital impact. Training in digital tools, specifically, increased the odds of a CHW’s use of a digital device. These findings are consistent with prior literature identifying the connection of training and improved digital device use [15, 25] as well as performance [26]. For example, a recent study in Kenya found that training and experience outperformed literacy and formal education as predictors of CHW knowledge and performance [26]. Though education and literacy are often used in the selection processes of CHWs globally, the link between these characteristics and CHW knowledge and performance are mixed [27–33]. Therefore, organizers and funders of CHW programs would benefit from supporting consistent, regular trainings specifically training in digital tools to encourage use of digital technologies in the CHW workforce.

### Barriers to digital access at the community level

The total number of barriers were not significantly associated with the current use of digital, but there was a positive relationship with believing in digital impact indicating that as the number of barriers to digital use increases, the greater impact a CHW thinks digital can have on their community. This relationship is contrary to what was anticipated but may suggest that, regardless of the barriers that CHWs identify for individuals in their community, belief in digital impact remains an important factor in aspiration for efficiency of health care delivery at the community level. A recently published systematic review identified that mobile health technologies can mitigate or even overcome current challenges experienced by CHWs in the promotion of health behaviors [15]. Another review also identified that willingness and perceived used of digital health technologies actively enables consistent and positive use of these technologies [25]. A similar relationship was discovered for the barrier of limited or no internet connectivity and the current use of digital devices. This may be because although CHWs use digital devices in the field, many digital applications operate as “offline-first” or do not require internet connectivity to operate effectively [11]. Therefore, limited or no internet connectivity is not viewed as a barrier to the use of digital devices and CHW programs should continue to use “offline-first” digital application models.

Out of the eight potential barriers to digital access for the community, two were significantly negatively associated with digital use: cost of phone/device and cost of mobile phone services. This finding illuminates an important distinction that must be made around cost and funding for CHWs and digital health. Our cost metrics were specific to the responsibility of individuals in a CHW’s community (which would include the CHW themselves) and not a government or organizational payer. The identification of cost as a barrier is consistent with results published in a recent systematic review which identified the cost of mobile phone service as a challenge of digitalization for CHW programs specific to the care and management of NCDs [9]. Purchasing a mobile phone data plan for the CHW’s own device has potential to be an effective approach to sustain the digital tool use and mitigate this personal barrier [34]. Sustainable funding of digital tools, like government- or organization-sponsored mobile phone data plans, is likely to be needed for organizations to continue to effectively operate and use digital devices within their community health workforce.

Another important result regarding the relationship between perceived barriers and digital use resides in the barriers that were not statistically significant. Limited experience or not knowing how to use technology, preferring traditional face-to-face interaction, and distrust in technology were not significantly related to digital use. These barriers were also not endorsed at the same level as the barriers related to cost when considering the descriptive statistics. These null results are important in understanding how to best prioritize which barriers to tackle as it relates to digital health access in communities and for CHWs. Therefore, future study around the barriers to digital access should focus on cost as the best approach to reduce the cost barrier for CHWs remains unknown.

### Increase competence through digital tools and motivating factors

CHWs who were optimistic about digital health, early adopters of digital in their personal lives, and found great value in their work recognized digital as helping them to have greater impact in their work in the community. Some of the most frequently endorsed impact items were improved and faster data collection, easy access to health education, and more frequent contact with community members without travel. The speed, ease, and convenience of digital is acknowledged as a way that CHWs can have impact with their communities. This was a significant finding given that the study sample was more tenured than not, and that their experiences with community health work as well as with digital has sustained this optimism. A scoping review identified that introductions to new technologies motivates CHWs and can be an overall enjoyable experience [15]. These positive experiences have been shown to also improve levels of self-efficacy and community recognition, giving CHWs a sense of pride and empowerment, and elevating their social status within their communities [15]. Positive beliefs and self-efficacy of digital technologies can be taught and further supported in trainings and community health work experience which has been demonstrated in prior work [15, 25]. In turn, these supports can be motivating to the CHW workforce and boost competence which was demonstrated during the COVID-19 pandemic [13]. A study done with female Health Extension Workers in Ethiopia indicated that a desire to help their community, recognition or respect gained from the community, and achievement were major motivating factors for competence and work performance [35]. Motivated and satisfied CHWs are likely to have better performance and retention than those that are not [35–37].

It is also important to note that there are several demotivating factors that can have an opposite effect, meaning less interest and optimism in digital technologies supporting and providing impact to a community. The same study by Eijigu et al. also indicated that inadequate pay and benefits, limited education and career advancement opportunities, workload, work environment, limited supportive supervision and absence of opportunity to change the workplace were demotivating factors [35]. CHWs are rarely paid for their services and are often grossly underpaid for their services [38]. Although we did not ask about their compensation, CHWs in the sample rarely identified supplementing their income as a value about their work. Yet, they are a critical piece to healthcare delivery across the world [39–41]. In June 2023, approximately 71 of 137 countries had one or more CHW groups that were accredited, but only half of these [35] were salaried [42]. Payment models considering country-specific legal frameworks within the context of the health system have been explored [38]. Nevertheless, CHWs are a workforce that health systems across the globe have begun to rely on yet they are not compensated adequately with salary, career advancement, and supportive supervision. By improving the working conditions of CHWs through adequate pay and resources, there is a potential to improve motivating factors which would ultimately translate into improved health outcomes in their communities.

### Age and tenure are not obstacles to utilizing digital in the community

Only two demographic factors were associated with the outcomes of this study. The oldest age category was significantly more likely to be a current digital device user when providing services to their community compared to 18–24-year-olds. We also discovered that working as a CHW for 1–5 years or more than 5 years, relative to less than one year, was associated with a greater belief in digital impact. This is inconsistent with prior work and sentiments that older age groups, or a more tenured workforce may be less engaged in digital technologies or resistant to changes especially as it relates to technology [43–45]. To the contrary, these results demonstrate that CHWs of older ages and longer tenure are engaging in digital and, therefore, that age and tenure should not be considered a hindrance to utilizing digital in the community health workforce.

### Strengths and limitations

Our study has several limitations. First, the online survey was disseminated widely across all regions where participation was voluntary and variable. Approximately 71% of CHWs in the sample were from Kenya and the Philippines. One of the many organizations that we contacted employs CHWs in Kenya and the Philippines and was a strong supporter and advocate for this project. Significant differences were found across these countries compared to each other and the total sample. This may be due to the payment methodologies or social structures within these specific regions. Despite these differences, it would be inappropriate to make inferences since this level of exploration is outside the scope of this project. Between and within country comparisons in CHW populations are recommended and should be explored in future work. In contrast, there was limited participation in specific areas where CHWs work including India, Brazil, and other regions in Latin America. Prior work shows that digital health technologies are being explored in these geographies and these models are supporting task extension of CHWs [46–48]. Second, using an online survey as a means for data collection may have biased the sample by selecting participants with some level of digital uptake and acceptance from the sampled CHWs. This approach, however, allowed for multi-country reach including language accessibility. Future research could address this limitation with a validation survey done by in-person interviews to overcome literacy and digital self-selection bias. Third, there was no inclusion/exclusion criteria for CHWs to participate outside of missing data; therefore, the CHWs represented are heterogeneous and represent a broad range of varied services provided, types of trainings, etc. However, the CHWs included in the study were well-tenured and well-trained which should be considered when interpreting results. Fourth, we initially limited CHWs to select up to three values when answering the question “What do you value most about your work?” to understand value priority in the sample. We then summed this variable from 0 to 3 to account for any confounding it may introduce in the final multinomial regression model while also avoiding over-fitting of the model by including all response options. However, there may be misrepresentation of total value in their work given that the respondents were only able to select up to three values for the item. Fifth, the proportional odds assumption was violated in an attempt to utilize an ordinal regression model for the sum of belief in digital impact outcome variable. Therefore, we selected a multinomial model to understand the relationship between training, barriers, and other covariates with the total for potential community impact.

## Conclusions

Inclusion and expansion of CHWs in health care teams is gaining momentum as a model for addressing the twin global health goals of having adequate health care worker capacity and decentralizing services to the community level. In this study, we assessed CHW experience with using digital health information tools to support their work. Our study suggests that CHWs are engaged and optimistic about use of digital technologies and their role in healthcare delivery. Trainings, specifically in digital tools, can increase a CHW’s use of digital devices as well as improve their beliefs in how digital can have impact in their community that ultimately can improve their competence. Ongoing concerns about cost of maintaining digital access in communities remain high; more research is needed to improve our understanding of how to reduce the cost barrier that CHWs face in their work with digital. Older age and greater tenure are not obstacles for digital use and belief in digital impact, respectively.

Collectively, CHWs are competent and trusted healthcare workers who have the potential to improve health outcomes and perhaps even reverse the global healthcare worker shortage. Governments and organizations that support digital CHW programs must reinforce motivating factors. Specifically, we recommend organizations provide CHWs with the appropriate digital resources and trainings, consider reducing personal cost barriers by subsidizing digital devices and services, and support CHWs’ values in their work. Through these calls to action, CHWs can achieve optimal task extension via digital technologies and elevate their self-efficacy, competence, and work performance. Digital technologies have the potential to shape the future of medical work. With the social capital and competence of community health workers, populations will engage and reap the benefits of digitization in healthcare.

### Supplementary Information

**Supplementary Material 1.**

## Data Availability

The dataset used and analyzed is available from the corresponding author on reasonable request.

## Abbreviations

AOR: Adjusted Odds Ratio
CHW: Community Health Worker
CI: Confidence Interval
LMIC: Low- and Middle-Income Country
OR: Odds Ratio
SD: Standard Deviation
NCD: Non-communicable disease
