# Supplementary material for: The use and potential impact of digital health tools at the community level: results from a multi-country survey of community health workers
Source: BMC Public Health. 2024 Mar 1;24:650. doi: 10.1186/s12889-024-18062-3 (PMC10905785; doi:10.1186/s12889-024-18062-3)
Supplement: Supplementary file 1 — Supplementary Material 1. [file 12889_2024_18062_MOESM1_ESM.docx]

Supplemental Material

| **Table 1. Summary of unadjusted bivariate associations with current digital use** | | | |
| --- | --- | --- | --- |
|  | **Total** | **Kenya** | **Philippines** |
|  | OR (95% CI) | OR (95% CI) | OR (95% CI) |
| *CHW trainings (sum)* | 1.02 (0.99-1.05) | 1.04 (0.99-1.08) | **1.18 (1.06-1.33)** |
| Digital tools training | **2.84 (2.06-3.98)** | **2.78 (1.84-4.27)** | **3.31 (1.36-9.90)** |
| *Barriers to the community (sum)* | 0.97 (0.90-1.04) | 1.06 (0.96-1.18) | 1.00 (0.81-1.27) |
| Limited or no internet connectivity | **1.62 (1.21-2.18)** | 1.39 (0.95-2.05) | 1.92 (0.97-3.78) |
| Limited or no electricity/power source | 1.04 (0.74-1.47) | 1.40 (0.93-2.12) | 1.29 (0.35-8.32) |
| Cost of mobile phone services | **0.62 (0.46-0.83)** | 0.85 (0.58-1.26) | 0.78 (0.40-1.53) |
| Cost of internet services | 1.04 (0.78-1.39) | **1.74 (1.17-2.56)** | 1.09 (0.54-2.27) |
| Cost of phone/device | **0.67 (0.50-0.90)** | 0.83 (0.56-1.22) | 1.02 (0.50-2.24) |
| Limited experience with technology/not knowing how to use technology | 0.81 (0.60-1.10) | 0.77 (0.52-1.14) | 0.94 (0.45-2.12) |
| Prefer traditional face-to-face interaction | 1.07 (0.77-1.50) | 1.38 (0.88-2.21) | 0.55 (0.27-1.19) |
| Distrust in technology | 0.92 (0.56-1.58) | 1.02 (0.53-2.07) | -- |
| No particular barriers | 1.16 (0.51-3.13) | 0.32 (0.06-1.44) | 0.59 (0.08-11.72) |
| Age (ref = 18-24 years)  25-34 years  35-44 years  45-54 years  55-64 years  65 years and over | 2.04 (0.89-4.38)  1.39 (0.61-2.92)  1.33 (0.58-2.83)  1.57 (0.65-3.66)  **4.36 (1.19-20.92)** | 1.94 (0.58-6.17)  1.45 (0.46-4.36)  1.91 (0.60-5.79)  1.90 (0.55-6.30)  **10.5 (1.44-217.8)** | --  --  --  --  -- |
| Gender (ref = Prefer not to say)  Female  Male  Non-binary | 2.03 (0.63-5.78)  2.18 (0.65-6.48)  1.00 (0.14-9.04) | 1.53 (0.39-5.47)  1.80 (0.44-6.70)  0.67 (0.06-7.55) | --  --  -- |
| Geographic region (ref = Suburban)  Rural  Urban | 0.93 (0.51-1.62)  0.98 (0.52-1.76) | 0.87 (0.39-1.82)  0.84 (0.36-1.83) | --  -- |
| CHW tenure (ref = Less than 1 year)  1-5 years  More than 5 years | 1.16 (0.60-2.13)  0.75 (0.40-1.30) | 3.16 (0.99-10.1)  2.25 (0.76-6.72) | 1.28 (0.27-4.43)  1.28 (0.28-4.34) |
| Note: **Bolded values** indicate statistical significance at the p < 0.05 level. “--“ represents a not significant relationship though the cell sizes were too small to accurately calculate an odds ratio. | | | |

| **Table 2. Summary of unadjusted bivariate associations with potential community impact** | | | | | | |
| --- | --- | --- | --- | --- | --- | --- |
|  | **Total** | | **Kenya** | | **Philippines** | |
|  | **Moderate Impact**  OR (95% CI) | **High Impact**  OR (95% CI) | **Moderate Impact**  OR (95% CI) | **High Impact**  OR (95% CI) | **Moderate Impact**  OR (95% CI) | **High Impact**  OR (95% CI) |
| *CHW trainings (sum)* | **1.19 (1.14-1.23)** | **1.34 (1.28-1.40)** | **1.24 (1.15-1.32)** | **1.42 (1.32-1.53)** | **1.14 (1.05-1.24)** | **1.31 (1.20-1.43)** |
| Digital tools training | **1.67 (1.22-2.28)** | **3.92 (2.83-5.43)** | **2.80 (1.56-5.05)** | **7.40 (4.03-13.57)** | 1.27 (0.69-2.33) | **3.11 (1.68-5.77)** |
| Current digital use | 0.96 (0.66-1.37) | 1.00 (0.68-1.47) | 1.07 (0.64-1.79) | 1.15 (0.67-1.99) | 1.66 (0.75-3.67) | 1.32 (0.58-3.00) |
| *Barriers to the community (sum)* | **1.75 (1.56-1.96)** | **2.35 (2.08-2.66)** | **2.31 (1.84-2.91)** | **3.40 (2.65-4.36)** | **1.73 (1.35-2.22)** | **2.53 (1.94-3.29)** |
| *Value about work (sum)* | **2.42 (1.96-3.00)** | **4.18 (3.11-5.63)** | **3.17 (2.15-4.69)** | **6.71 (3.69-12.21)** | **2.42 (1.65-3.55)** | **3.02 (1.89-4.85)** |
| Optimism in digital  (ref = neutral)  Not at all optimistic  Not optimistic  Optimistic  Very optimistic | 0.45 (0.15-1.36)  0.95 (0.32-2.78)  **1.59 (1.03- 2.47)**  **2.32 (1.53- 3.51)** | --  --  **2.27 (1.32-3.92)**  **5.31 (3.19-8.85)** | 0.35 (0.07-1.79)  --  1.21 (0.52-2.78)  1.23 (0.60-2.55) | 0.30 (0.03-3.05)  --  1.71 (0.64-4.59)  **2.88 (1.22-6.82)** | --  --  1.67 (0.84-3.32)  1.79 (0.87-3.69) | --  --  **2.31 (1.00-5.34)**  **3.39 (1.45-7.93)** |
| Digital adoption  (ref = wait for others)  First to try new tech  Last to try new tech | **1.72 (1.22-2.41)**  1.18 (0.58-2.43) | **2.31 (1.58-3.37)**  1.57 (0.73-3.39) | 1.75 (0.86-3.53)  1.20 (0.36-3.92) | **2.39 (1.08-5.27)**  1.73 (0.48-6.18) | 0.95 (0.53-1.68)  0.79 (0.25-2.53) | 1.18 (0.63-2.23)  0.61 (0.15-2.56) |
| Age (ref = 18-24 years)  25-34 years  35-44 years  45-54 years  55-64 years  65 years and over | 1.73 (0.49-1.83)  1.93 (0.84-3.56)  1.46 (0.95-3.93)  2.03 (0.89-4.63)  0.85 (0.31-2.32) | 23.4 (3.06-179.04)  21.0 (2.75-160.74)  22.9 (2.99-175.45)  35.3 (4.44-280.18)  12.0 (1.38-104.79) | 0.97 (0.29-3.31)  1.43 (0.45-4.59)  1.45 (0.45-4.70)  1.91 (0.48-7.58)  1.67 (0.24-11.59) | --  --  --  --  -- | 1.64 (0.27-9.79)  2.45 (0.42-14.18)  1.42 (0.24-8.38)  2.01 (0.31-12.96)  1.00 (0.11-8.99) | --  --  --  --  -- |
| Gender (ref = Prefer not to say)  Female  Male  Non-binary | 0.75 (0.18-3.01)  0.95 (0.23-3.96)  1.00 (0.06-15.99) | 0.54 (0.13-2.20)  0.85 (0.20-3.54)  1.50 (0.11-21.31) | --  --  -- | --  --  -- | --  --  -- | --  --  -- |
| Geographic region (ref = Suburban)  Rural  Urban | 1.02 (0.55-1.88)  1.17 (0.62-2.21) | 1.17 (0.32-1.03)  0.63 (0.34-1.18) | 1.50 (0.59-3.76)  1.32 (0.50-3.46) | 1.06 (0.42-2.64)  0.85 (0.32-2.23) | 0.30 (0.03-2.92)  0.29 (0.03-2.90) | 0.29 (0.03-3.27)  0.42 (0.04-4.82) |
| CHW tenure (ref = Less than 1 year)  1-5 years  More than 5 years | **2.34 (1.38-3.98)**  **2.61 (1.58-4.32)** | **5.19 (2.54-10.59)**  **5.60 (2.81-11.17)** | **7.50 (2.09-26.87)**  **6.49 (1.97-21.41)** | --  -- | 1.79 (0.58-5.49)  **3.08 (1.04-9.16)** | **9.75 (1.22-77.99)**  **9.47 (1.19-75.20)** |
| Note: Reference for the outcome level was low impact (0-2). **Bolded values** indicate statistical significance at the p < 0.05 level. “--“ represents a not significant relationship though the cell sizes were too small to accurately calculate an odds ratio. | | | | | | |

| **Table 3. Summary of adjusted multivariate associations with current digital use** | | | |
| --- | --- | --- | --- |
|  | **Total** | **Kenya** | **Philippines** |
|  | AOR (95% CI) | AOR (95% CI) | AOR (95% CI) |
| *CHW trainings (sum)* | -- | -- | **1.14 (1.02-1.30)** |
| Digital tools training | **2.92 (2.09-4.13)** | **2.54 (1.67-3.94)** | 2.26 (0.88-7.02) |
| Limited or no internet connectivity | **1.62 (1.19-2.20)** | -- | -- |
| Cost of mobile phone services | **0.68 (0.49-0.95)** | -- | -- |
| Cost of internet services | -- | 1.46 (0.96-2.22) | -- |
| Cost of phone/device | **0.66 (0.47-0.92)** | -- | -- |
| Age (ref = 18-24 years)  25-34 years  35-44 years  45-54 years  55-64 years  65 years and over | 1.86 (0.79-4.14)  1.41 (0.60-3.08)  1.39 (0.59-3.07)  1.63 (0.65-3.93)  **4.73 (1.26-23.21)** | 1.46 (0.43-4.80)  1.07 (0.33-3.34)  1.35 (0.41-4.25)  1.20 (0.36-4.51)  7.14 (0.94-150.62) | -- |
| Note: **Bolded values** indicate statistical significance at the p < 0.05 level. “--“ represents a not significant relationship though the cell sizes were too small to accurately calculate an odds ratio. | | | |

| **Table 4. Summary of adjusted multivariate associations with potential community impact** | | | | | | |
| --- | --- | --- | --- | --- | --- | --- |
|  | **Total** | | **Kenya** | | **Philippines** | |
|  | **Moderate Impact**  AOR (95% CI) | **High Impact**  AOR (95% CI) | **Moderate Impact**  AOR (95% CI) | **High Impact**  AOR (95% CI) | **Moderate Impact**  AOR (95% CI) | **High Impact**  AOR (95% CI) |
| Digital tools training | **1.46 (1.03-2.07)** | **3.03 (2.04-4.49)** | **2.32 (1.15-4.67)** | **5.83 (2.71-10.25)** | 1.25 (0.63-2.47) | **2.95 (1.40-6.22)** |
| *Barriers to the community (sum)* | **1.60 (1.42-1.80)** | **2.10 (1.85-2.39)** | **2.15 (1.69-2.75)** | **3.08 (2.36-4.03)** | **1.54 (1.19-1.99)** | **2.22 (1.67-2.93)** |
| *Value about work (sum)* | **2.03 (1.61-2.55)** | **3.07 (2.18-4.32)** | **2.85 (1.79-4.55)** | **5.48 (2.67-10.12)** | **2.14 (1.44-3.18)** | **2.49 (1.44-4.31)** |
| Optimism in digital  (ref = neutral)  Not at all optimistic  Not optimistic  Optimistic  Very optimistic | 0.38 (0.12-1.22)  0.92 (0.26-3.22)  1.47 (0.90-2.40)  **1.88 (1.18-3.01)** | 0.19 (0.02-1.75)  --  **2.08 (1.08-3.99)**  **3.87 (2.09-7.18)** | 0.33 (0.05-2.06)  1.06 (0.11-9.79)  1.15 (0.42-3.20)  1.36 (0.56-3.33) | 0.36 (0.02-5.77)  --  1.44 (0.04-5.15)  **3.15 (1.03-9.64)** | --  --  1.69 (0.80-3.57)  1.77 (0.81-3.89) | --  --  2.39 (0.90-6.33)  **3.19 (1.18-8.62)** |
| Digital adoption  (ref = wait for others)  First to try new tech  Last to try new tech | **1.75 (1.19-2.58)**  1.30 (0.58-2.91) | **2.27 (1.41-3.64)**  2.13 (0.82-5.58) | 1.66 (0.69-3.99)  2.34 (0.53-10.30) | 2.14 (0.73-6.30)  **6.15 (1.04-36.37)** | -- | -- |
| CHW tenure (ref = Less than 1 year)  1-5 years  More than 5 years | **2.30 (1.26-4.19)**  **2.22 (1.26-3.91)** | **5.06 (2.15-10.19)**  **4.25 (1.86-9.71)** | **9.39 (1.92-45.90)**  **6.12 (1.40-26.65)** | --  -- | 2.11 (0.63-1.07)  2.76 (0.86-8.89) | **10.78 (1.20-96.60)**  7.69 (0.87-67.83) |
| Note: Reference for the outcome level was low impact (0-2). **Bolded values** indicate statistical significance at the p < 0.05 level. “--“ represents a not significant relationship though the cell sizes were too small to accurately calculate an odds ratio. | | | | | | |
